# Machine learning to identify risk factors associated with the development of ventilated hospital-acquired pneumonia and mortality: implications for antibiotic therapy selection

**DOI:** 10.3389/fmed.2023.1268488

**Published:** 2023-12-15

**Authors:** Anthony Sophonsri, Mimi Lou, Pamela Ny, Emi Minejima, Paul Nieberg, Annie Wong-Beringer

**Affiliations:** ^1^Department of Clinical Pharmacy, University of Southern California, Mann School of Pharmacy and Pharmaceutical Sciences, Los Angeles, CA, United States; ^2^Department of Pharmacy, Huntington Hospital, Pasadena, CA, United States; ^3^Department of Pharmacy, Los Angeles General Medical Center, Los Angeles, CA, United States; ^4^Department of Medicine – Infectious Diseases, Huntington Hospital, Pasadena, CA, United States

**Keywords:** pneumonia, critical care, mechanical ventilation, multidrug resistance, machine learning

## Abstract

**Background:**

Among patients with nosocomial bacterial pneumonia, those who decompensated to requiring mechanical ventilation (vHABP) faced the highest mortality followed by ventilator-associated pneumonia (VABP) and non-ventilated hospital-acquired pneumonia (nvHABP). The objectives of this study were to identify risk factors associated with the development and mortality of vHABP and to evaluate antibiotic management.

**Methods:**

A multicenter retrospective cohort study of adult inpatients with nosocomial pneumonia during 2014–2019 was performed. Groups were stratified by vHABP, nvHABP, and VABP and compared on demographics, clinical characteristics, treatment, and outcomes. Multivariable models were generated via machine learning to identify risk factors for progression to vHABP as well as pneumonia-associated mortality for each cohort.

**Results:**

457 patients (32% nvHABP, 37% vHABP, and 31% VABP) were evaluated. The vHABP and nvHABP groups were similar in age (median age 66.4 years) with 77% having multiple comorbidities but more vHABP patients had liver disease (18.2% vs. 7.7% *p* = 0.005), alcohol use disorder (27% vs. 7.1%, *p* < 0.0001), and were hospitalized within the past 30  days (30.4% vs. 19.5%, *p* = 0.02). An immediate need for ventilatory support occurred in 70% of vHABP patients on the day of diagnosis. Mortality was the highest in vHABP followed by VABP and nvHABP groups (44.6% vs. 36% vs. 14.3%, *p* < 0.0001). Nearly all (96%) vHABP patients had positive cultures, with Gram-negative pathogens accounting for 58.8% whereby 33.0% were resistant to extended-spectrum β-lactams (ESBLs), ceftriaxone (17.5%), fluoroquinolones (20.6%), and carbapenems (12.4%). Up to half of the vHABP patients with ESBL-Enterobacterales or *P. aeruginosa* did not receive an effective empiric regimen; over 50% increase in mortality rate was observed among patients whom effective therapy was initiated past the day of pneumonia diagnosis. Risk factors associated with vHABP development were alcohol use disorder, APACHE II score, vasopressor therapy prior to infection, and culture positive for ESBL-Enterobacterales whereas history of hospitalization in the past 30  days, active malignancy, isolation of ceftriaxone-resistant pathogens or *Pseudomonas aeruginosa*, and vasopressor therapy were risk factors for vHABP-associated mortality.

**Conclusion:**

Patients with vHABP experienced an acute and severe decompensation upon diagnosis. The risk factors identified in this study could provide actionable data for clinicians to identify those at risk for vHABP at the onset of pneumonia and to target antimicrobial stewardship efforts to improve treatment success.

## Background

Nosocomial pneumonia (NP) is a leading hospital-acquired infection that accounts for 22% of cases and is associated with prolonged hospitalization and significant mortality (1). Nosocomial pneumonia may be grouped into three subtypes: ventilator-associated bacterial pneumonia (VABP), and ventilated (vHABP) and non-ventilated hospital-acquired bacterial pneumonia (nvHABP) (2, 3). VABP accounts for roughly half of the cases of NP while the remaining half is equally divided between nvHABP and vHABP (4). Up to 40% of mechanically ventilated patients develop VABP with an all-cause mortality risk of 20%–50% (5, 6). Thus, concerted efforts have focused on preventive measures to target reduction of VABP occurrence to zero (7).

Although HABP is generally considered less severe, more than 50% of patients develop serious complications including respiratory failure, septic shock, and empyema (8). Notably, mortality has been shown to be highest for HABP patients who progress to vHABP compared to VABP and nvHABP (4, 6, 9). A recent multicenter retrospective study using administrative data to compare the epidemiology and clinical outcome of patients with nvHABP, vHABP, and VABP found that more vHABP patients required ICU admission and vasopressor therapy, had a prolonged hospitalization, and were more likely to be discharged to hospice among survivors. In another single-center retrospective study, Motowski et al. compared patients with ventilated pneumonias (vHABP vs. VABP) and similarly found that vHABP was associated with significantly higher 30-day and in-hospital all-cause mortality and longer length of stay (10). The growing evidence surrounding vHABP-associated morbidity and mortality supports further investigation to identify risk factors associated with the development of vHABP and death in order to facilitate early recognition of at-risk individuals and to help guide antibiotic management.

Recently, machine learning (ML) has been adopted into medical research as a method of minimizing bias and improving the accuracy of predictive models. ML is a branch of artificial intelligence that applies statistical techniques to produce a trained model fitted to a given data set. Among the ML algorithms, random forests are an increasingly popular statistical method of classification and regression. Random forests are a combination of tree predictors such that each tree depends on the values of random vectors sampled independently and with the same distribution for all trees in the forest (11). Few studies have applied machine learning to predict risk of developing pneumonia, but have not explored risk factors associated with poor outcomes including disease progression and mortality (12, 13). Thus, our study objectives were to identify risk factors prior to or at onset of HABP diagnosis associated with progression to vHABP and mortality and to evaluate empiric antibiotic management using a machine learning approach.

## Materials and methods

### Study population and design

This was a retrospective cohort study conducted at two sites: Huntington Hospital and Los Angeles General Medical Center-University of Southern California. This study was conducted in accordance with the amended Declaration of Helsinki. The study protocol was approved by the institutional review boards (IRB) at both centers (Advarra IRB Pro00045861; University of Southern California IRB: HS-20-00663). Informed consent was waived.

Eligible patients were hospitalized adults (≥18 years) who developed NP between March 2014 and December 2019. Hospitalized patients with a secondary diagnosis of pneumonia ICD-9 and ICD-10 codes were screened for inclusion; those with a primary diagnosis of community-acquired pneumonia were excluded. Pneumonia diagnosis was confirmed with documentation of new or progressive radiographic infiltrate in addition to clinical findings suggestive of infection such as new-onset fever, purulent sputum, leukocytosis, and decline in oxygenation (14). Pregnant patients and patients with pneumonia of non-bacterial etiology were excluded. Hospital-acquired pneumonia was defined as pneumonia developing >48 h from admission. Non-ventilated HABP (nvHABP) was defined as HABP without the need for endotracheal intubation but allowing for use of non-invasive ventilation (e.g., nasal cannula, high flow nasal cannula, bi-level positive airway pressure, etc.) during the course of infection whereas ventilated HABP (vHABP) was defined as HABP subsequently requiring endotracheal intubation at any time during the course of infection (including at onset). Ventilator-associated pneumonia was defined as pneumonia developing >48 h after endotracheal intubation (2). Due to a significantly higher proportion of patients with nvHABP at one study site, patients who met inclusion criteria for nvHABP were randomly selected to achieve a relatively balanced distribution across the 3 groups.

### Clinical evaluation

Patients’ medical records were reviewed for pertinent demographic, laboratory, and clinical information as follows: age, gender, comorbid conditions, social history, residence prior to admission, receipt of immunosuppressive therapy, hospitalization within the past 30 days or receipt of antibiotics within the past 90 days, severity of illness (Acute Physiology and Chronic Health Evaluation, APACHE II score), intensive care unit (ICU) admission, and need for and duration of vasopressor therapy and mechanical ventilation, vital signs, daily labs, culture and sensitivity results, clinical management (oxygen supplementation, antibiotic therapy), and outcomes (hospital and ICU lengths of stay and all-cause in-hospital mortality).

### Study definitions and endpoints

The APACHE II score was calculated at onset of pneumonia diagnosis. Empiric therapy was defined as any antibiotic administered prior to or without knowledge of pathogen identity and/or susceptibility. Effective therapy was any antibiotic regimen containing at least one agent with documented *in vitro* activity against the isolated pathogen from the respiratory culture. The primary endpoints were risk of development of vHABP and in-hospital mortality. Study data were managed using REDCap, a secure web-based platform designed for data capture in research studies (15).

### Data analysis

Patients were grouped by subtypes of NP (vHABP, nvHABP, and VABP). Our primary analysis was to compare those who developed vHABP vs. nvHABP on demographics, comorbidities, and clinical and microbiological features at time of pneumonia diagnosis as well as empiric treatment to identify predisposing risk factors for developing vHABP and vHABP-associated mortality. The VABP group was included for relative comparison. Descriptive analysis was performed using Mann Whitney U or Student t-test for continuous variables and chi-square or Fisher’s exact test for categorical variables where appropriate. Odds ratio (OR) with 95% confidence intervals (CI) were calculated. A modified Poisson regression analysis using error variance was used to analyze time to receipt of effective therapy to identify the incremental risk for in-hospital mortality with day 0 (i.e., effective therapy started before or on the day of respiratory culture was taken) as the reference group. A supervised machine learning algorithm, the Random Forests (RF) method, was employed in this study. Breiman in 2001 defined a random forest as a classifier consisting of a collection of tree-structured classifiers {h(x, ϴ_k_), k = 1, …} where the {ϴ_k_} are independently and identically distributed random vectors and each tree casts a unit vote for the most popular class at input x. For the k^th^ tree, a random vector ϴ_k_ is generated, independent of the past random vectors ϴ_1_, …, ϴ_k-1_ but with the same distribution (11). The strength of the individual trees in the forest and the correlation between them determines the generalization error of a forest of the tree classifier. Combining trees grown using random features can produce improved accuracy (11). Rodriguez-Galiano et al. provided a flowchart illustration of the RF method (16). The RF method performs both classification and regression prediction. It enables a more robust, accurate, and stable prediction than the Classification and Regression Trees (CART) by building multiple decision trees and merging the predictions by averaging the posterior probabilities for interval targets or voting for class targets (17). A SAS High Performance procedure, HPFOREST, was applied, to create random forest models in a high performance environment. The data was split proportionally into a training set [i.e., input data or inBag fraction (16)] and “out-of-bag” (OOB) data to measure the accuracy of the model and reduce the misclassification rate. The training set for a tree was a sample without replacement from all available observations. Averaging over trees from different training samples reduced the dependence of the predictions on any particular training sample. The OOB sample, a set of observations not used in building the current tree, was used to estimate the prediction error, evaluate variable importance, and monitor correlation (11, 16). The difference between the misclassification rate for the modified and original OOB data divided by the standard error determined the importance of the variable ranked from most to least important (17). About 50 clinical factors assessed as continuous or categorical variables with the potential to impact primary or secondary outcomes were selected as the input to the random forest ensembles which included age, gender, race, APACHE-II, Charlson Comorbidity Index, alcohol use disorder, malignancy, liver disease and 14 other frequently occurring comorbid conditions, receipt of vasopressor therapy, isolation of *P. aeruginosa*, resistance phenotype of the respiratory pathogen, ICU admission prior to pneumonia diagnosis, and empiric antibiotic therapy. About 14–18 preselected factors in the random forest method were then included in the logistic regression forward variable selection one-by-one in the order of their importance (i.e., ranking). The area under the receiver operating characteristic (ROC) curve was estimated and compared to assess which factors were highly influential in the model prediction. Those factors were then further explored using a backwards selection logistic regression model. The interaction effects were not included. The final multivariable logistic regression models only included significant predictors for the major endpoints: risk of development of vHABP and pneumonia-associated-mortality. All variables that had less than 5% of values missing were included as candidates in the machine learning-based models (18). All statistical tests were 2-tailed and a *p*-value < 0.05 was considered significant. Statistical analyses were performed using SAS software, version 9.4 (SAS Institute Inc., Cary, NC, United States).

## Results

### Study population

A total of 457 patients were included, aiming for a relatively balanced distribution of patients into the 3 subtypes of NP (32.4% vHABP, 37.0% nvHABP, and 30.6% VABP). Nearly all patients across the 3 groups were admitted for a non-infectious diagnosis (vHABP: 89.9%; nvHABP: 89.3%; VABP: 93.6%) of which the most common were conditions related to the respiratory tract (16.1%, *n* = 67) followed by central nervous system (14.2%, *n* = 59), and gastrointestinal tract (10.8%, *n* = 45).

Baseline demographics were similar between vHABP and nvHABP groups. Patients included in the study were from a diverse population comprised of 40.0% Latino, followed by 24.3% White, 12.9% Black, and 9.6% Asian. Median age was 66.4 years (IQR: 56.6, 76.9) with most patients being male (65.4%) and presented from home (74.2%). Patients in the vHABP group were predominantly Latino (39.2% vs. 27.2%) whereas patients in the nvHABP group were predominantly White (38.5% vs. 24.3%). Compared to the nvHABP group, more patients with vHABP had ≥3 comorbidities (82.4% vs. 72.2%, *p* = 0.03), particularly, a history of liver disease (18.2% vs. 7.7%, *p* = 0.005) and alcohol use disorder (27% vs. 7.1%, *p* < 0.0001) (Table 1). Interestingly, risk factors for multidrug resistance including immunosuppression (16.2% vs. 23.7%, *p* = 0.10) and 90-day antibiotic history (5.4% vs. 3%, *p* = 0.27) were similar between the two groups except for a higher prevalence of hospitalization in the prior 30 days (30.4% vs. 19.5%, *p* = 0.02) in the vHABP group compared to the nvHABP group. In contrast, the VABP group tended to be younger (median age: 57.1 years, IQR: 42.7, 63.8) and less likely to have ≥3 comorbidities (60%). Similar to the vHABP group, patients with VABP were predominantly Latino (56.4%) with a notable history of alcohol use disorder (22.1%) and liver disease (16.4%).

**Table 1:** Baseline characteristics.

Characteristic	All patients (*n* = 457)	vHABP (*n* = 148)	nvHABP (*n* = 169)	VABP (*n* = 140)	*p*-value^a^
Age, median years (IQR)	62.7 (53.2–73.1)	65.5 (55.6–74.0)	68.0 (57.2–79.7)	57.1 (42.7–63.8)	0.09
Male, *n* (%)	299 (65.4)	98 (66.2)	97 (57.4)	104 (74.3)	0.11
**Race/ethnicity, *n* (%)**					<0.01
Asian	44 (9.6)	18 (12.2)	16 (9.5)	10 (7.1)	
Black	59 (12.9)	16 (10.8)	30 (17.8)	13 (9.3)	
Latino	183 (40)	58 (39.2)	46 (27.2)	79 (56.4)	
White	111 (24.3)	36 (24.3)	65 (38.5)	10 (7.1)	
Others	60 (13.1)	20 (13.5)	12 (7.1)	28 (20.0)	
**Residence, *n* (%)**					0.07
Home	339 (74.2)	107 (72.3)	139 (82.3)	93 (66.5)	
LTAC/SNF	18 (3.9)	7 (4.7)	8 (4.7)	3 (2.1)	
Other	100 (21.9)	34 (23.0)	22 (13.0)	44 (31.4)	
**Comorbidities, *n* (%)**					
Neurologic disorder	110 (24.1)	44 (29.7)	39 (23.1)	27 (19.3)	0.18
Diabetes mellitus	148 (32.4)	54 (36.5)	50 (29.6)	44 (31.4)	0.19
Chronic lung conditions	93 (20.4)	33 (22.3)	37 (21.9)	23 (16.4)	0.93
COPD	35 (7.7)	10 (6.8)	18 (10.7)	7 (5.0)	0.22
ILD	12 (2.6)	5 (3.4)	6 (3.6)	1 (0.7)	>0.99
Cardiovascular diseases	292 (63.9)	104 (70.3)	118 (69.8)	70 (50.0)	0.93
Liver disease	63 (13.8)	27 (18.2)	13 (7.7)	23 (16.4)	0.005
Renal impairment	96 (21)	36 (24.5)	43 (25.4)	17 (12.1)	0.85
Malignancy	80 (17.5)	31 (20.9)	38 (22.5)	11 (7.9)	0.74
3+ comorbidities	328 (71.8)	122 (82.4)	122 (72.2)	84 (60.0)	0.03
CCI, median (IQR)	4 (2–6)	5 (3–7)	4 (3–6)	2 (1–5)	0.12
**Social history, *n* (%)**					
Active smoker	71 (15.5)	28 (18.9)	21 (12.4)	22 (15.7)	0.11
Alcohol use disorder	83 (18.2)	40 (27.0)	12 (7.1)	31 (22.1)	<0.0001
IVDU	47 (10.3)	9 (6.1)	8 (4.7)	30 (21.4)	0.60
**Risk for multidrug resistance, *n* (%)**					
Immunocompromised	73 (16)	24 (16.2)	40 (23.7)	9 (6.4)	0.10
Prior hospitalization within 30 days	96 (21)	45 (30.4)	33 (19.5)	18 (12.9)	0.02
Prior antibiotics within 90 days	14 (3.1)	8 (5.4)	5 (3.0)	1 (0.7)	0.27
**Admitting diagnosis, *n* (%)**					
Infection-related (non-pneumonia)	42 (9.2)	15 (10.1)	18 (10.7)	9 (6.4)	0.88
Non-infection related	415 (90.8)	133 (89.9)	151 (89.3)	131 (93.6)	
Respiratory	67 (14.7)	29 (21.8)	17 (11.3)	21 (16.0)	
Central nervous system	59 (12.9)	15 (11.3)	8 (5.3)	36 (27.5)	
Gastrointestinal	45 (9.8)	17 (12.8)	22 (14.6)	6 (4.6)	

CCI, Charlson comorbidity index; IVDU, intravenous drug use; LTAC, long-term acute care facility; SNF, skilled nursing facility; Chronic lung conditions included asthma, bronchiectasis, chronic obstructive pulmonary disease (COPD), interstitial lung disease (ILD), obstructive sleep apnea (OSA), pulmonary tuberculosis (TB), or requiring supplemental oxygen at home; Other under residence included outside hospital, homelessness, or jail.

a *p*-values are for the comparison of vHABP and nvHABP groups only.

### Clinical characteristics and outcomes

Pneumonia onset from admission was a median of 6 days for both the vHABP and nvHABP groups (Table 2). Notably, the majority of patients in the vHABP group (70.3%, 104/148) had an immediate need for ventilatory support, occurring on the day of diagnosis [median 0 days (IQR: 0, 1)], suggesting an acute and severe decompensation. There was also a higher prevalence of ICU admission (52% vs. 27.8%, *p* < 0.0001) and vasopressor use prior to pneumonia diagnosis (48.6% vs. 4.7%, *p* < 0.0001) in the vHABP compared to the nvHABP group. Additionally, patients in the vHABP group had a significantly higher APACHE II score (median 25.0 vs. 12.0, *p* < 0.0001) on the day of pneumonia diagnosis compared to the nvHABP group. Overall, significantly more vHABP patients required ICU level of care (100% vs. 37.9%, *p* < 0.0001) with over half requiring vasopressor therapy (57.4% vs. 6.5%, *p* < 0.0001) during infection when compared to the nvHABP group (Table 2). Patients with vHABP had worse outcomes than those with nvHABP: longer post-infection ICU stay (median 10 d vs. 3 d, *p* < 0.0001) and overall length of stay (median 24 d vs. 13.5 d, *p* < 0.0001), and 3-fold higher in-hospital mortality rate (44.6% vs. 14.3%, *p* < 0.0001). Compared to the vHABP group, patients in the VABP group had similar severity of underlying illness (median APACHE II score 23.5). Despite the VABP group requiring a longer duration of ICU stay (12.5 d vs. 10 d) and prolonged duration of mechanical ventilation (8 d vs. 6 d), in-hospital mortality (36% vs. 44.6%) remained lower than those with vHABP.

**Table 2:** Clinical characteristics and outcome.

Characteristic	All patients (*n* = 457)	vHABP (*n* = 148)	nvHABP (*n* = 169)	VABP (*n* = 140)	*p*-value^a^
Pneumonia diagnosis from time of admission, median days (IQR)	6.0 (3.0–10.0)	6.0 (3.0–11.0)	6.0 (4.0–10.0)	6.0 (3.5–9.0)	0.86
**Severity of illness**					
APACHE II, median (IQR)	20.0 (14.0–26.0)	25.0 (19.0–32.0)	12.0 (8.0–18.0)	23.5 (19.0–28.0)	<0.0001
ICU prior to infection, *n* (%)	263 (57.5)	77 (52.0)	47 (27.8)	139 (99.3)	<0.0001
Vasopressor therapy prior to infection, *n* (%)	126 (27.6)	72 (48.6)	8 (4.7)	46 (32.9)	<0.0001
**Clinical course of pneumonia**					
ICU during infection, *n* (%)	350 (76.6)	148 (100)	64 (37.9)	139 (99.3)	<0.0001
Post-infection ICU LOS, median days (IQR)	10.0 (5.0–18.0)	10.0 (5.0–17.0)	3.0 (2.0–5.0)	12.5 (8.0–22.0)	<0.0001
Vasopressor therapy during infection, *n* (%)	142 (31.1)	85 (57.4)	11 (6.5)	46 (32.9)	<0.0001
Duration of MV during infection, median days (IQR)	7.0 (4.0, 12.0), *n* = 154	6.0 (4.0–11.0), *n* = 133		8.0 (3.0–12.0), *n* = 121	0.62^b^
**Outcome**	(*n* = 455)	(*n* = 148)	(*n* = 168)	(*n* = 139)	
Hospital LOS, median days (IQR)	35.0 (17.0–38.0)	24.0 (16.0–39.0)	13.5 (9.0–23.0)	27.0 (18.0–37.0)	<0.0001
In-hospital mortality, *n* (%)	140 (30.8)	66 (44.6)	24 (14.3)	50 (36.0)	<0.0001

APACHE II, Acute Physiology and Chronic Health Evaluation II; ICU, intensive care unit; LOS, length of stay; MV, mechanical ventilation.

a *p*-values are for the comparison of vHABP and nvHABP groups only.

b *p*-value for comparison between vHABP and VABP groups.

### Microbiology

A greater number of patients in the vHABP group had a positive respiratory culture compared to the nvHABP group (96.1% vs. 75%, *p* < 0.0001) (Table 3). Infectious etiology among culture-positive patients was similar between the vHABP and nvHABP groups (monomicrobial: 71.7% vs. 68.8%, *p* = 0.71; gram-negative: 58.8% vs. 45.7%, *p* = 0.26). Notably, the vHABP group had numerically higher prevalence of cetriaxone-resistant Enterobacterales (17.5% vs. 13%, *p* = 0.50) and resistance to extended-spectrum ß-lactams (33% vs. 23.9%, *p* = 0.27) and carbapenems (12.4% vs. 8.7%, *p* = 0.58) than the nvHABP group. With respect to the VABP group, all patients had a positive culture. One third of the isolated pathogens were resistant to extended-spectrum ß-lactams and fluoroquinolones.

**Table 3:** Microbiology and empiric therapy.

Characteristic	All patients (*n* = 457)	vHABP (*n* = 148)	nvHABP (*n* = 169)	VABP (*n* = 140)	*p*-value^a^
**Respiratory culture, *n* (%)**	307 (67.2)	103 (69.6)	64 (37.9)	140 (100)	<0.0001
Culture positive	287/307 (93.5)	99/103 (96.1)	48/64 (75)	140/140 (100)	<0.0001
Monomicrobial	182 (63.4)	71 (71.7)	33 (68.8)	78 (55.7)	0.71
Polymicrobial	105 (36.6)	28 (28.3)	15 (31.2)	62 (44.3)	
**Pathogen, *n* (%)**	(*n* = 282)	(*n* = 97)	(*n* = 46)	(*n* = 139)	0.26
Gram-positive	76 (27.0)	29 (29.9)	16 (34.8)	31 (22.3)	
Gram-negative	152 (53.9)	57 (58.8)	21 (45.7)	74 (53.2)	
Mixed	54 (19.1)	11 (11.3)	9 (19.6)	34 (24.5)	
**Resistance phenotype, ***n*** (%)**	(*n* = 282)	(*n* = 97)	(*n* = 46)	(*n* = 139)	
Ceftriaxone^b^	57 (20.2)	17 (17.5)	6 (13.0)	34 (24.5)	0.50
Extended spectrum ß-lactam^c^	88 (31.2)	32 (33.0)	11 (23.9)	45 (32.4)	0.27
Carbapenem	24 (8.5)	12 (12.4)	4 (8.7)	8 (5.8)	0.58
Fluoroquinolones^d^	76 (27.0)	20 (20.6)	10 (21.7)	46 (33.1)	0.88
**Empiric therapy**					
**Agent(s) prescribed, *n* (%)**	(*n* = 455)	(*n* = 148)	(*n* = 168)	(*n* = 139)	
Azithromycin	36 (7.9)	11 (7.4)	21 (12.5)	4 (2.9)	0.14
Ceftriaxone (3^rd^ generation)	86 (18.9)	30 (20.3)	26 (15.5)	30 (21.6)	0.27
β-lactam agents with antipseudomonal activity	288 (63.3)	107 (72.3)	124 (73.8)	57 (41.0)	0.76
Ceftazidime or cefepime	121 (26.6)	48 (32.4)	38 (22.6)	35 (25.2)	0.05
Piperacillin-tazobactam	153 (33.6)	54 (36.5)	77 (45.8)	22 (15.8)	0.09
Carbapenem	49 (10.8)	24 (16.2)	22 (13.1)	3 (2.2)	0.43
Vancomycin	239 (52.5)	90 (60.8)	84 (50.0)	65 (46.8)	0.05
Metronidazole	78 (17.1)	41 (27.7)	19 (11.3)	18 (12.9)	0.0002
**Receipt of effective regimens by select pathogens**^**f**^					
MRSA, *n* (%)	28/47 (62.2)	12/15 (80)	6/10 (60)	10/22 (45.5)	0.38
ESBL-Enterobacterales, *n* (%)	15/35 (42.9)	8/13 (61.5)	2/5 (40.0)	5/17 (29.4)	0.61
*Pseudomonas aeruginosa, n (%)*	24/51 (47.1)	10/19 (52.6)	7/8 (87.5)	7/24 (29.2)	0.19
**Time to receipt of effective regimen**					
Less than 48 h after diagnosis, *n* (%)	200/285 (70.2)	67/99 (67.7)	37/47 (78.7)	96/139 (69.1)	0.17

a *p*-values are for the comparison of vHABP and nvHABP groups only.

b Ceftriaxone-resistance only includes Enterobacterales in which susceptibility was performed.

c Extended spectrum ß -lactams include ceftriaxone, cefepime, and/or piperacillin-tazobactam.

d Fluoroquinolones include ciprofloxacin and levofloxacin.

f Regimens containing at least one agent with documented in vitro activity against the organism(s) isolated for patients with culture-positive pneumonia.

### Antimicrobial therapy

The most common agents prescribed for empiric therapy were antipseudomonal β-lactams (63.3%, 288/455) followed by vancomycin (52.5%, 239/455), ceftriaxone (18.9%, 86/455), metronidazole (17.1%, 78/455), and azithromycin (7.9%, 36/455) (Table 3). Choice of empiric agents was similar between groups except for higher utilization of vancomycin (60.8% vs. 50%, *p* = 0.05), antipseudomonal cephalosporins (32.4% vs. 22.6%, *p* = 0.05), and metronidazole (27.7% vs. 11.3%, *p* = 0.0002) in the vHABP compared to the nvHABP group. The most common agents among the VABP group were vancomycin (46.8%) and antipseudomonal cephalosporins (25.2%).

Among those with a positive respiratory culture, the proportion of patients receiving an effective empiric regimen in the vHABP group was 80% for MRSA; however, nearly half of the patients with ESBL-Enterobacterales (39.5%) and *P. aeruginosa* (47.4%) did not receive effective empiric therapy. In comparison, a higher proportion of patients in the nvHABP group (87.5%) with *P. aeruginosa* received an effective empiric regimen. Overall, patients with vHABP were less likely to receive an effective regimen within 48 h of pneumonia diagnosis compared to those with nvHABP (67.7% vs. 78.7%, *p* = 0.17), though the difference was not statistically significant. Importantly, mortality risk increased by 1.55 fold (95% CI, 0.98–2.46, *p* = 0.06) for those who received effective empiric therapy 1–2 days after the day of diagnosis (Table 4). Despite lower rates of effective empiric regimens against MRSA (45.5%), ESBL-Enterobacterales (29.4%), and *P. aeruginosa* (29.2%), the overall mortality rate was lower in the VABP than vHABP group.

**Table 4:** Relative risk of in-hospital mortality by time of delay to effective therapy for vHABP patients with positive cultures (*n* = 99).

Time to receipt of effective therapy	Death, *n* (%^a^)	Mortality rate, %	Relative risk (95% CI)	*p*-value^b^
Day 0 (Started before or on the day of respiratory culture was taken), *n* = 55	21 (38.2)	1.8	Reference	N/A
Started Day 1 or Day 2 after respiratory culture was taken, *n* = 27	16 (59.3)	3.7	1.55 (0.98–2.46)	0.06
Started Day 3 or later after respiratory culture was taken, *n* = 17	7 (41.2)	5.9	1.08 (0.56–2.09)	0.82

^a^Row percentage in each category of time to receipt of effective therapy.

b *p*-value associated with relative risk.

### Machine learning-derived multivariable models

**Figure 1:**
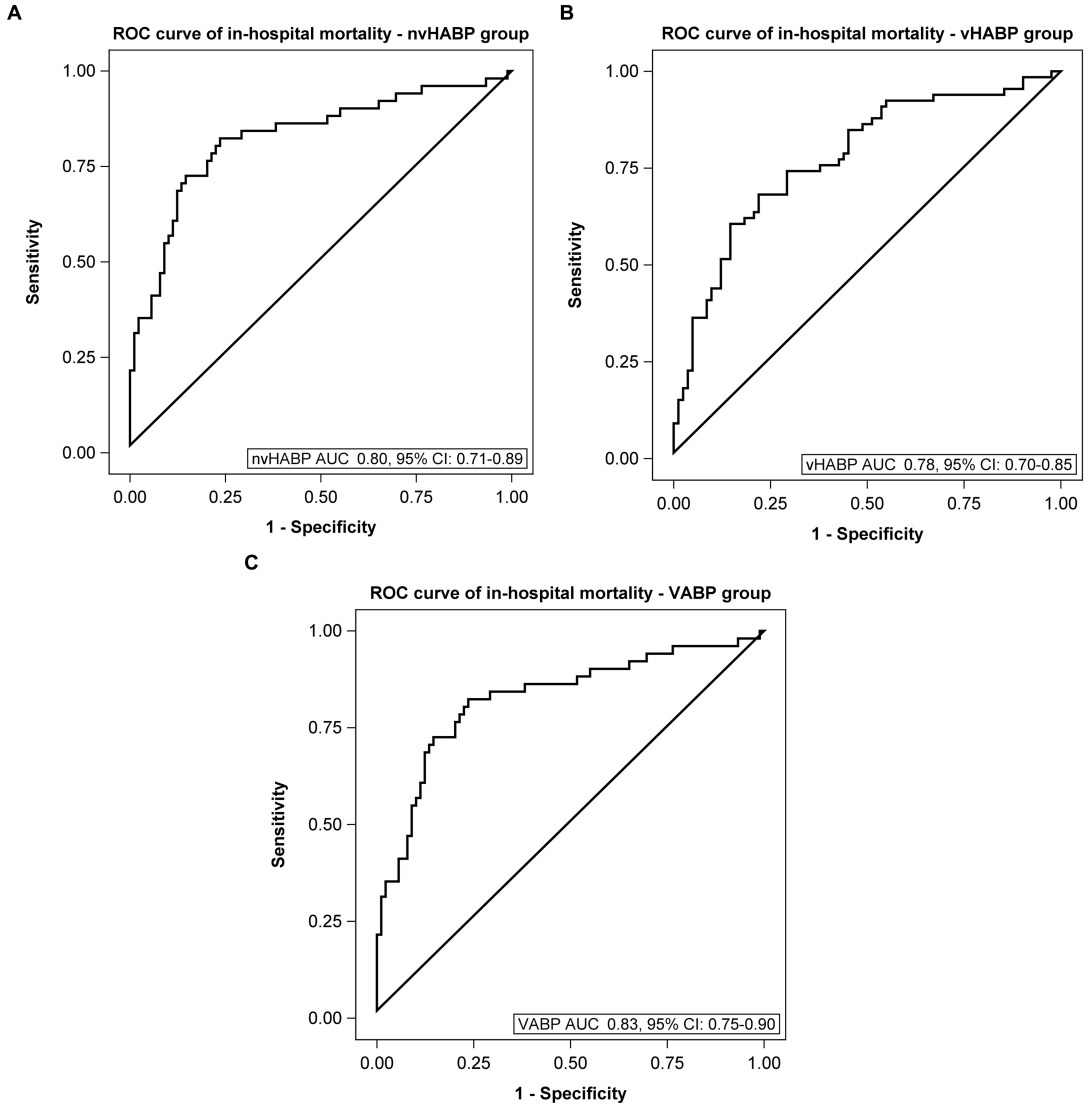
Area under the receiver operating characteristic curve of mortality models. **(A)** Non-ventilated, hospital-acquired bacterial pneumonia (nvHABP), **(B)** ventilated, hospital-acquired bacterial pneumonia (vHABP), **(C)** ventilator-associated bacterial pneumonia (VABP). AUC, area under the curve; ROC, receiver operating characteristic.

We applied machine learning to develop multivariable models for identifying potential risk factors for the development of vHABP (Table 5) and pneumonia-associated-mortality, controlling for age (Table 6). The following factors were significantly associated with the development of vHABP: alcohol use disorder (OR 3.49, 95% CI: 1.40 to 8.67; *p* = 0.007), APACHE II score at time of pneumonia diagnosis (OR 1.19, 95% CI: 1.14 to 1.25; *p* < 0.0001), isolation of ESBL-pathogens (OR 3.35, 95% CI: 1.37 to 8.20; *p* = 0.008), and vasopressor therapy prior to infection (OR 6.91, 95% CI: 2.84 to 16.79; *p* < 0.0001). Among those who developed vHABP, risk factors associated with mortality include isolation of ceftriaxone-resistant pathogens (OR 3.24, 95% CI: 0.96 to 1.01; *p* = 0.04) or *P. aeruginosa* (OR 3.08. 95% CI: 097 to 9.72; *p* = 0.06), active malignancy (OR 4.33, 95% CI: 1.64 to 11.4; *p* = 0.003), prior hospitalization within 30 days (OR 2.16, 95% CI: 0.96 to 4.86; *p* = 0.06), and vasopressor therapy during infection (OR 2.83, 95% CI: 1.31 to 6.13; *p* = 0.01) (Table 6). Similarly, active malignancy (OR 4.24, 95% CI: 0.97 to 18.51; *p* = 0.05), and vasopressor therapy during infection (OR 12.62, 95% CI: 5.15 to 30.95; *p* < 0.0001) were identified as significant predictors of mortality in the VABP group. Additionally, isolation of ESBL-pathogens (OR 2.76, 95% CI: 1.11 to 6.89; *p* = 0.03) was another risk factor for mortality identified in the VABP group. In contrast, risk factors identified in the nvHABP group were age (OR 1.04, 95% CI 1.01 to 1.09; *p* = 0.02) APACHE II score (OR 1.16, 95% CI: 1.08 to 1.25; *p* = 0.0001) and empiric vancomycin therapy (OR 2.94, 95% CI: 1.06 to 8.17; *p* = 0.04) (Table 6). The AUC ROC of the nvHABP, vHABP, and VABP mortality models were 0.80 (95% CI 0.71 to 0.89), 0.78 (95% CI 0.70 to 0.85), and 0.83 (95% CI 0.75 to 0.90), respectively (Figure 1).

**Table 5:** Predictors of vHABP development from multivariable analysis.

Development of vHABP (*N* = 317)	Odds ratio (95% CI)	*p*-value
Alcohol use disorder	3.49 (1.40–8.67)	0.007
APACHE II score	1.19 (1.14–1.25)	<0.0001
ESBL (phenotypic resistance)	3.35 (1.37–8.20)	0.008
Vasopressor therapy prior to infection	6.91 (2.84–16.79)	<0.0001

APACHE II, Acute Physiology and Chronic Health Evaluation II; ESBL, Extended-spectrum β-lactamase.

**Table 6:** Predictors of in-hospital mortality from multivariable analyses.

Variable	Odds ratio (95% CI)	*p*-value
**vHABP (***n*** = 148)**		
Age	0.99 (0.96–1.01)	0.26
Ceftriaxone resistance	3.24 (1.02–10.26)	0.04
Isolation of *P. aeruginosa*	3.08 (0.97–9.72)	0.06
Malignancy	4.33 (1.64–11.4)	0.003
Prior hospitalization within 30 days	2.16 (0.96–4.86)	0.06
Vasopressor therapy during infection	2.83 (1.31–6.13)	0.01
**VABP (***n*** = 169)**		
Age	1.03 (0.99–1.06)	0.10
ESBL (phenotypic resistance)	2.76 (1.11–6.89)	0.03
Malignancy	4.24 (0.97–18.51)	0.05
Vasopressor therapy during infection	12.62 (5.15–30.95)	<0.0001
**nvHABP (***n***= 140)**		
Age	1.04 (1.01–1.08)	0.02
APACHE II score	1.16 (1.08–1.25)	0.0001
Empiric vancomycin therapy	2.94 (1.06–8.17)	0.04

ESBL, Extended-spectrum B-lactamase; APACHE II, Acute Physiology and Chronic Health Evaluation II.

## Discussion

This is a retrospective cohort analysis of patients with HABP differentiated into nvHABP and vHABP to determine risk factors associated with the development of vHABP and vHABP-associated mortality using a machine learning approach. The advantage of using the random forest algorithm in machine learning over traditional methods to identify predictive risk factors is that the former yields improved accuracy and precision while minimizing bias, supporting its use as a promising alternative to traditional predictive tools (19–22). Although risk factors for vHABP-associated mortality and morbidity have been identified previously, our study provides unbiased confirmation for several known as well as newly identified risk factors associated with immune-disrupting chronic conditions and antimicrobial resistance. Consistent with prior published literature, our findings also confirm that vHABP is associated with significantly worse outcomes than either nvHABP or VABP. Importantly, our study provides actionable information prior to onset of pneumonia that could facilitate early recognition of those at risk for developing vHABP and potential treatment considerations to improve outcomes.

Overall, baseline characteristics were similar among study groups. One notable exception when comparing between vHABP and nvHABP groups is that a significantly greater proportion of the vHABP group had liver disease (18.2% vs. 7.7%, *p* = 0.005) and alcohol use disorder (27% vs. 7.1%, *p* < 0.0001). It is well established that patients with severe liver disease have compromised immune function thereby increasing the risk and severity of infection (23). Pneumonia is a frequent complication particularly among patients with cirrhosis (24, 25). Additionally, several studies have linked alcohol use disorder with poor outcomes among patients with community-acquired pneumonias (26–28). Both liver disease and alcohol use disorder were independently associated with poor outcomes in this study. However, only alcohol use disorder was selected by the Random Forest algorithm as it is likely the stronger predictor of mortality compared to liver disease despite significant correlation between the two factors as determined by *post hoc* analysis. Notably, the vHABP group experienced significantly worse outcomes compared to the nvHABP group: longer post-infection ICU stay (median 10 vs. 3d, *p* < 0.0001), higher utilization of vasopressors during infection (57.4% vs. 6.5%, *p* < 0.0001), longer length of hospital stay (24 vs. 13.5d, *p* < 0.0001), and higher in-hospital mortality (44.6 vs. 14.3%, *p* < 0.0001). Although duration of mechanical ventilation, ICU stay, and hospitalization were relatively longer in the VABP compared to the vHABP group, the latter had numerically higher mortality rate (44.6% vs. 36%, *p* = 0.12). We speculate that mortality among vHABP patients may be attributable in part to advanced age coupled with a lower immunological reserve for containing the infection among those with underlying liver disease or alcohol use disorder. Additionally, malignancy and hospitalization within the past 30 days were significant risk factors associated with mortality in patients with vHABP. Most patients with malignancies are immunocompromised as a result of disease or chemotherapy and are at risk for poor outcomes, especially infection-related mortality (29). Furthermore, patients with recent hospitalization are at increased risk of acquiring multi-drug resistant infections in which the probability of receiving initial ineffective therapy is high. Multiple studies have shown that delays in effective therapy negatively impacted outcomes including length of stay and survival among patients with multi-drug resistant Enterobacterales and *P. aeruginosa* (30–32).

More vHABP patients were hospitalized in the 30 days before admission compared to nvHABP patients (30.4% vs. 19.5%, *p* = 0.02). Accordingly, culture positivity was nearly 2-fold higher in the vHABP compared to the nvHABP group with a numerically higher prevalence of ceftriaxone-, carbapenem-, and ESBL-resistant phenotypes. To our knowledge, this is the first study comparing resistance phenotypes across the three different classifications of nosocomial pneumonia. The major pathogens of concern among culture-positive patients with vHABP were *P. aeruginosa*, ESBL-Enterobacterales, and *S. aureus*. As confirmed by our machine learning-derived multivariable model, isolation of an ESBL-producing organism was a significant predictor for vHABP development (OR 3.35, 95% CI: 1.37 to 8.2; *p* = 0.008) while isolation of *P. aeruginosa* (OR 3.08, 95% CI: 0.97 to 9.72; *p* = 0.06) and ceftriaxone resistance (OR 3.24, 95% CI: 1.02 to 10.26; *p* = 0.04) was associated with vHABP-associated mortality.

With respect to empiric therapy, more patients with MRSA isolation received an effective regimen (vHABP: 80%, nvHABP: 60%, and VABP: 45.5%) compared to those with isolation of ESBL-Enterobacterales or *P. aeruginosa*. It is notable that patients with nvHABP receiving empiric vancomycin therapy had nearly 3-fold higher risk of mortality (OR 2.94, 95% CI: 1.06 to 8.17; *p* = 0.04) which could potentially serve as a surrogate marker for a subpopulation with more complex underlying disease in whom broad antimicrobial coverage was initiated. For patients with vHABP involving ESBL-Enterobacterales, nearly 40% did not receive an effective empiric regimen. In addition, despite the high rate of empiric antipseudomonal coverage in all 3 groups, nearly half of the patients (47.4%) with vHABP involving *P. aeruginosa* did not receive an effective empiric regimen. Considering that 70% of our vHABP group experienced an acute rapid respiratory decompensation requiring ventilatory support within 24 h of pneumonia diagnosis, prompt initiation of an effective empiric regimen is of paramount importance. As expected, delays in receipt of effective therapy significantly increased the risk of mortality. For patients in the vHABP group, we observed over 50% increase in mortality rate when effective therapy was not initiated on or before the day of pneumonia diagnosis. Given the global concern of rising multidrug resistance, our findings underscore the need to provide empiric coverage that encompasses ESBL-producing organisms and *P. aeruginosa* in patients at risk for developing vHABP considering the high prevalence of recent healthcare exposure in this subpopulation.

Our study had several limitations. First, our cohort may be subject to selection bias. Patients were initially screened based on ICD-9 and ICD-10 codes. Although all related codes were included in the screening criteria, there may be patients with nosocomial pneumonia that were missed in the initial screening. Second, this was a retrospective study conducted over a 5-year period at 2 different institutions. We acknowledge that the standard of care may have changed over the study period and that practice standards may differ between the two study sites. Notably, cefepime and piperacillin-tazobactam are differentially preferred as empiric agents of choice at the two institutions; however, both agents empirically cover *P. aeruginosa* (risk factor for vHABP-associated mortality) and the choice of agent was not identified as a significant risk factor for mortality on the multivariable model. Rather, resistance against either agent such as with ESBL-producing organisms was a significant predictor for development of vHABP which may contribute to the negative consequences from the delayed receipt of effective therapy. As the aim of this study was to identify predisposing or early risk factors that would distinguish at-risk patients for developing vHABP, we did not report on definitive therapy since by the time culture and sensitivities were reported, patients had already progressed to needing ventilatory support in the vHABP group. Lastly, we acknowledge that the current study represents an initial derivation study and that our models have not been externally validated which is necessary to confirm our results.

Our study further confirms the increase in morbidity and mortality associated with vHABP from previous studies. While the nvHABP and vHABP groups differed in various aspects of patient characteristics, clinical presentation, and microbiology based on univariate analysis, only a handful of variables were identified as factors significantly associated with the development of vHABP and vHABP-mortality in our machine learning-derived multivariable models. The novelty of our model removes the bias associated with *p*-value based selection methods that have been commonly utilized in prior studies. Importantly, the risk factors identified in our study provide actionable data for clinicians to identify those at risk for vHABP at the onset of pneumonia and to target antimicrobial stewardship efforts to improve treatment success.

## Conclusion

Taken together, alcohol use disorder, APACHE II score at pneumonia diagnosis, isolation of ESBL-producing pathogens, and need for vasopressor therapy prior to infection were risk factors associated with the development of vHABP. Among those who developed vHABP, prior hospitalization within the past 30 days, active malignancy, isolation of *P. aeruginosa* or ceftriaxone-resistant pathogens, and vasopressor therapy during infection increased the risk of death after controlling for age. As such, patients who have any of these risk factors should be monitored closely and have a lower threshold for escalation of therapy. Considering that isolation of a ceftriaxone-resistant organism or *P. aeruginosa* carries a risk for vHABP or in-hospital mortality respectively, it may be prudent to initiate empiric therapy against those organisms in patients who developed nosocomial pneumonia and require vasopressors, were recently hospitalized, or had a history of malignancy. Although these factors were identified by a machine learning derived model, external validation is needed to confirm the reliability of our results in real-world applications.

## Data availability statement

The raw data supporting the conclusions of this article will be made available by the authors, without undue reservation.

## Ethics statement

The studies involving humans were approved by Advarra IRB (Study No: Pro00045861) and University of Southern California IRB (Study No: HS-20-00663). The studies were conducted in accordance with the local legislation and institutional requirements. The ethics committee/institutional review board waived the requirement of written informed consent for participation from the participants or the participants' legal guardians/next of kin because this was a retrospective study.

## Author contributions

AS: Conceptualization, Data curation, Formal analysis, Investigation, Methodology, Writing – original draft, Writing – review & editing. ML: Formal analysis, Writing – review & editing. PNy: Data curation, Writing – review & editing. EM: Data curation, Writing – review & editing. PNi: Investigation, Supervision, Writing – review & editing. AW-B: Conceptualization, Formal analysis, Funding acquisition, Investigation, Methodology, Project administration, Resources, Supervision, Writing – review & editing.
